# Ultrasensitive optical detection of Fe^3+^ ions using fluorescent carbon quantum dots derived from Simmondsia Chinensis Jojoba leaves

**DOI:** 10.1038/s41598-025-25214-x

**Published:** 2025-11-18

**Authors:** M. Ghali, W. S. Mabior, H. Soliman, M. Sami, M. K. Elnimr

**Affiliations:** 1 Institute of Basic and Applied Science, Egypt-Japan University of Science and Technology, New Borg El-Arab City, Alexandria 21934 Egypt; 2 Pharmacognosy Department, Faculty of Pharmacy, Helwan University, Ain-Helwan, Cairo 11795 Egypt; 3 PharmD Program, Egypt-Japan University of Science and Technology, New Borg El-Arab City, Alexandria 21934 Egypt; 4 Physics Department, Faculty of Science, Tanta University, Tanta, Egypt; 5 Physics Department, Faculty of Science, Kafr Elsheikh University, Kafr Elsheikh, Egypt

**Keywords:** Simmondsia Chinensis (Jojoba), Fluorescent carbon quantum dots, Fe^3+^ ions detection, Materials science, Materials for devices

## Abstract

We report for the first time the synthesis of carbon quantum dots (CQDs) derived from Simmondsia chinensis (jojoba) leaves using a hydrothermal method at 180 °C for 10 h for ultrasensitive detection of Fe^3+^ ions in aqueous solutions. The obtained CQDs have an average size of 3.5 nm and exhibit stable blue fluorescence, making them suitable for optical sensing applications. They show remarkable selectivity toward Fe^3+^ ions and achieve an ultralow detection limit of 0.018 µM, outperforming most reported CQD-based sensors. The fluorescence quenching behavior follows the Stern–Volmer relation and confirms a static quenching mechanism between Fe^3+^ ions and surface functional groups of the CQDs. These results highlight the potential of jojoba-derived CQDs as low-cost, sustainable, and highly effective fluorescent probes for Fe^3+^ ion detection, offering a promising route for environmental monitoring applications.

## Introduction

Over the past two decades, extensive research has focused on fabricating Carbon Quantum Dots (CQDs) from natural, green, and renewable carbon precursors as an alternative to traditional organic dyes and semiconductor quantum dots^1–3^. These CQDs, a novel type of carbon-based nanomaterial, have shown considerable promise due to their unique optical, electronic, and chemical properties^4^. Researchers have defined them as carbon-based nanomaterials with sizes below 10 nm. CQDs were first discovered by Xu in 2004 during the purification of single-walled carbon nanotubes^5^, with their existence later confirmed by Sun in 2006^6^. Since their discovery, various techniques have been extensively reported for producing fluorescent CQDs, including hydrothermal synthesis, chemical oxidation, arc discharge, laser ablation, and microwave-assisted methods, among others^7^. Currently, the hydrothermal approach is the most widely adopted due to its cost-effectiveness, non-toxicity, environmental friendliness, and precise control over CQD properties. This method has been widely employed to synthesize CQDs from various natural precursors such as orange juice^8^, watermelon peels^9^, and banana peels^10^. These biomass-derived sources offer several advantages over traditional quantum dot precursors, including natural abundance, low cost, non-toxicity, and environmental friendliness, making them attractive green alternatives for sustainable CQD synthesis^11^.

CQDs are appealing for a wide range of applications owing to several intriguing properties^12^. Their powerful and adjustable photoluminescence, which allows them to emit varying colors of light based on surface chemistry and size, is one of their most remarkable characteristics^6^. Consequently, they are attractive candidates for many applications such as, light-emitting diodes^13^, supercapacitors^14^, solar cells, bioimaging, cell imaging^15^, and especially as fluorescence-based sensors of a wide range of analytes. For instance, CQDs have been employed in the detection of amino acids, food additives, and pharmaceutical residues due to their high sensitivity, low toxicity, and tunable photoluminescence properties^16,17^.

Interestingly, CQDs synthesized from various plant sources such as Borassus flabellifer^18^, Mangifera indica leaves^19^, betel leaf (Piper betel)^20^, and zedoary (Curcuma zedoaria)^21^ have demonstrated effective detection of metal ions, particularly Fe^3+^ ions^22^. While Fe^3+^ is an essential trace element for both humans and aquatic organisms, its excessive presence in the environment can cause severe ecological imbalance and significant health risks^23^. Therefore, the development of reliable strategies for monitoring Fe^3+^ is of critical importance. Notably, plant-derived CQDs typically exhibit a high degree of surface functionalization, enriched with diverse groups such as hydroxyl, carboxyl, and amino functionalities^24^. These groups play a crucial role in dictating the surface chemistry of CQDs, where oxygen- and nitrogen-containing moieties facilitate strong coordination with metal ions and promote electron transfer from photoexcited CQDs to Fe^3+^, resulting in efficient fluorescence quenching^25^.

Jojoba (Simmondsia Chinensis) plant of the Simmondsiaceae family, is a perennial shrub plant that is indigenous to the arid regions of northern Mexico and the southwestern United States. Jojoba has thick, leathery leaves and is a dioecious plant, meaning it has male and female flowers on separate plants^26^. Female plants produce fruit with one seed that looks somewhat like an acorn, known as a nut^27^. The Jojoba plant has become an essential plant around the world due to its medicinal properties, which have made it to be cultivated in many countries, including Chile, Argentina, Tunisia, Saudi Arabia, and Egypt^28^. It is highly prized for its seeds, which yield a special oil known as “liquid wax”. It’s referred to as liquid wax rather than oil because it contains mainly fatty acid ethers, sterols, and vitamins, with only small amounts of triglyceride esters^29^, giving the jojoba plant various potential applications in cosmetics, antifungal and anti-microbial, dermatology, and skin care activities^30^. However, this work highlights the potential of utilizing jojoba leaves for the sustainable synthesis of carbon quantum dots, which may open new avenues for their eco-friendly production. The primary objective of using jojoba leaves is to address their status as a discarded waste product with a lack of potential applications, thereby reducing organic waste accumulation in the environment. To this end, we report the first green synthesis of fluorescent CQDs derived from jojoba leaves and their application as an optical sensor for the ultrasensitive detection of Fe^3+^ ions in aqueous solution.

## Experimental details

### Materials

Jojoba leaves were collected from a local farm in Egypt (Wadi Natrun Farm, Egypt). The salts of different metal ions, which include Co(NO_3_)_2_.6H_2_O, CuSO_4_.5H_2_O, MgSO_4_.7H_2_O, CaCl_2_, FeCl_3_, (Ni(NO_3_)_2_.6H_2_O), MnCl_2_.6H_2_O, and ZnCl_2_, from Fisher Scientific UK, Deionized water was used throughout the experiment.

### Synthesis method

**Fig. 1:**
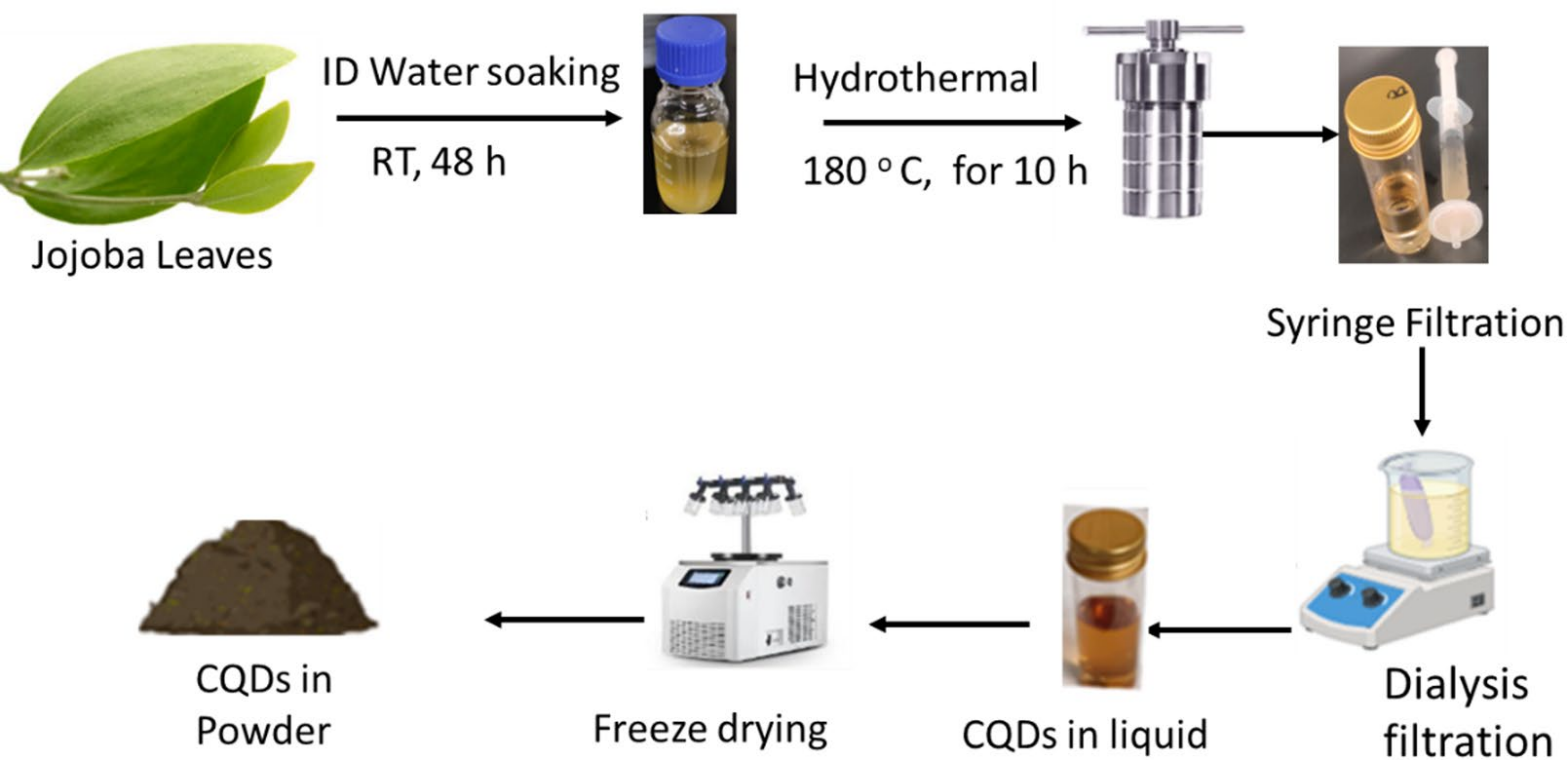
Schematic diagram for the synthesis of CQDs from jojoba leaves.

The experimental preparation was described as shown in schematic Fig. 1. The jojoba leaves were washed thoroughly using tap and deionized water to remove any dust particles and other impurities. Clean leaves were subsequently scratched and soaked in 100 mL of deionized water as a solvent for extraction within two days at room temperature. The extract was filtered with standard double-ring filter paper, then 30 mL of the extracted sample was transferred into a 50 mL Teflon-lined stainless-steel autoclave and heated in an oven at 180 °C for 10 h. The reaction was left to cool to room temperature and filtered again using both double rings filter paper followed by a syringe filter (0.22 µm). A light brown aqueous solution was obtained, and the carbon dots were collected using 1000 Da dialysis membrane against DI water for 48 h to get purified CQDs which are free from low-molecular-weight impurities and fluorophores that could otherwise interfere with their optical properties or sensing performance. The resultant supernatant was stored at 4 °C, which later lyophilized for further characterization.

### Characterization instruments

A transmission electron microscope (TEM) (JEM-2100F from JEOL Company, Japan, operated at 200 kV) was used to study the morphology of the formed CQDs. The samples for analysis were prepared on a copper grid coated with carbon and allowed to dry at room temperature. Bruker AVANCE HD III Nuclear Magnetic Resonance (NMR), operated at 400 MHz (1H NMR) and 100.40 MHz (^13^C NMR). The solvent used for compound 1 was DMSO. Fourier Transform Infrared spectroscopy (FTIR), VERTEX 70v from Bruker Company, was used to determine the surface functional group of the produced CQDs. FS5 Spectrofluorometer (Edinburgh Instrument) was used to analyze fluorescence intensity and fluorescence lifetimes of the synthesized CQDs. Hitachi U-3900 spectrophotometer was used to measure the UV–visible spectra of the synthesized CQDs. X-ray photoelectron spectroscopy (XPS) (Thermo Fisher Scientific, USA) was also used to investigate the chemical composition, elemental states, and electronic states of synthesized CQDs, and Malvern zeta sizer nano zeta potential was used to evaluate the stability and surface charge of synthesized CQDs.

### Sensing of Fe^3+^ ions

To evaluate the metal ion selectivity of the synthesized CQDs, fluorescence intensity was monitored as a function of wavelength in the presence of various metal ions. In details, 50 µL of different metal ion solutions (4 µM), including Co(NO_3_)_2_·6H_2_O, CuSO_4_·5H_2_O, MgSO_4_·7H_2_O, CaCl_2_, FeCl_3_, Ni(NO_3_)_2_·6H_2_O, and MnCl_2_·6H_2_O, were added to 2 mL of the as-prepared CQD solution. Among all tested metal ions, Fe^3+^ exhibited the most pronounced quenching effect, resulting in the lowest fluorescence intensity of the CQDs. To further investigate the sensitivity of CQDs towards Fe^3+^ ions, fluorescence measurements were performed using the same stock solution (4 µM). Incremental additions of 5 µL of the Fe^3+^ solution were made to 2 mL of CQDs, and the fluorescence intensity was recorded after each addition. The results clearly demonstrated a progressive decrease in fluorescence intensity with increasing Fe^3+^ ion concentration, confirming the strong quenching effect of Fe^3+^ on the CQDs.

### Measurement of fluorescence quantum yield (QY)

The fluorescence quantum yield (QY) of the synthesized CQDs was determined using an absolute method with an FS-5 Spectrofluorometer equipped with an SC-30 Integrating Sphere (Edinburgh Instruments). In this approach, the integrating sphere captures both the excitation light (transmitted and scattered) and the total emitted fluorescence. The QY is then calculated based on the ratio of the number of emitted photons to the number of absorbed photons, with the absorption derived from the difference between the incident and transmitted excitation light. Using this method, the QY of the CQDs was determined to be 3.35%.

## Results and discussion

### Morphological and structural properties of CQDs

**Fig. 2:**
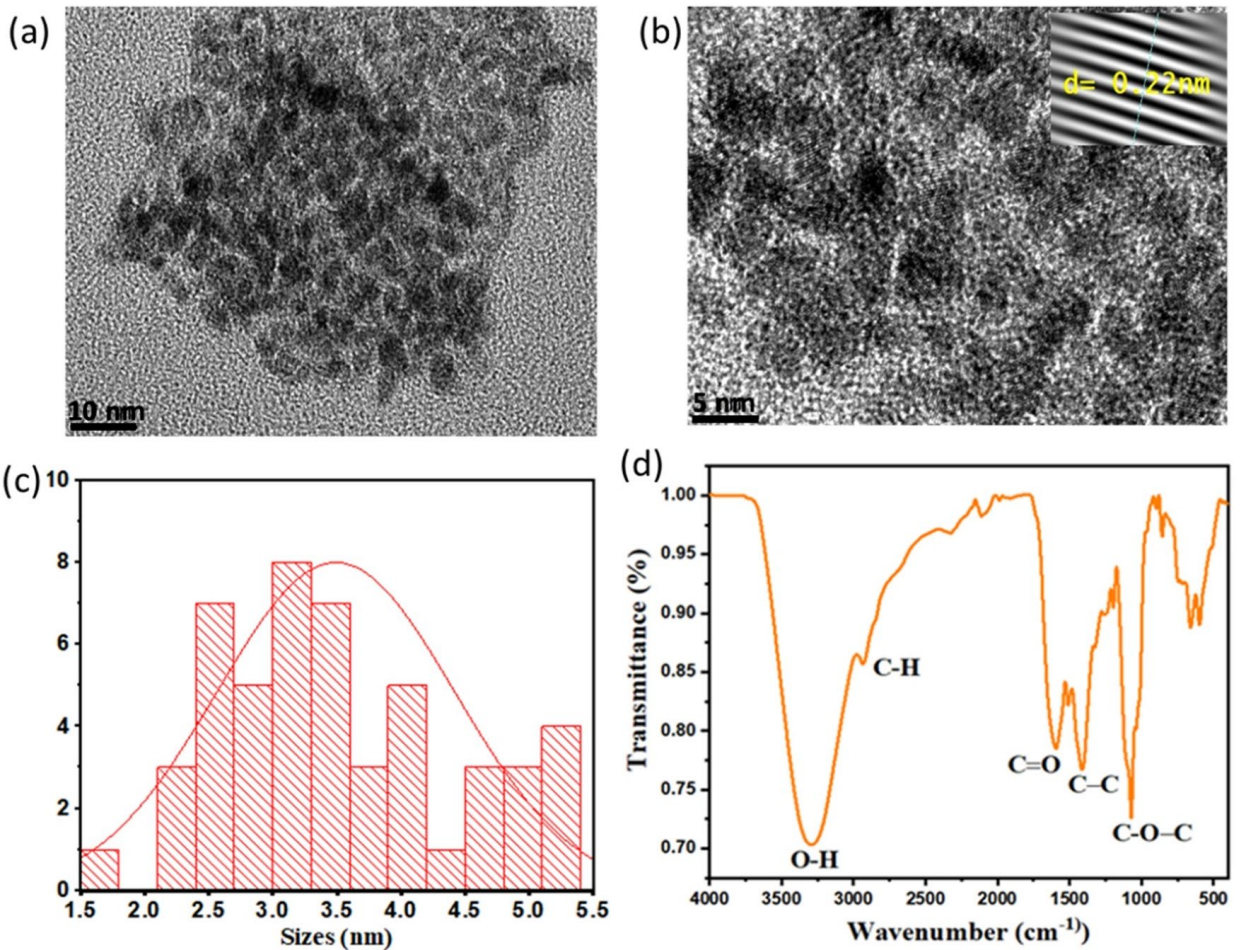
(**a**, **b**) TEM and HRTEM images of the synthesized CQDs, (**c**) dots size distribution. (**d**) FTIR spectrum of CQDs.

Transmission Electron Microscopy (TEM) was employed to analyze the morphology of the CQDs derived from jojoba leaves, as presented in Fig. 2a,b. These images illustrate well dispersed, quasi-spherical nanoparticles, surrounded by an amorphous carbon matrix^28^. The particle size distribution histogram in Fig. 2c indicates an average diameter of 3.50 nm, calculated from 90 particles. Furthermore, Fig. 2(b; inset) reveals well-defined lattice fringes consistent with a graphitic structure, with an interplanar spacing of 0.22 nm, as highlighted in the inset, in agreement with previous findings^31^.

Fourier Transform Infrared spectroscopy (FTIR) was employed to determine the surface functional groups of the as-prepared CQDs. The surface of Jojoba leaves synthesized CQDs indeed indicate various oxygenated groups, as indicated by the FTIR spectrum as seen in Fig. 2d. Namely, the FTIR spectrum reveals the presence of O–H stretching vibrations at 3296 cm^−1^ with broad high peak intensity, the weak absorption peak that occurs at 2932 cm^−1^ is assigned to C–H stretching vibration^32^. The peak observed at 1600 cm^−1^ is ascribed to carbon-to-oxygen double bond C=O , which may be related to the presence of minor impurities. The peak at 1397 cm^−1^ can be due to the presence of C–C, while the stretching vibrations peak at 1068 cm-1 is related to C–O–C functional groups of ether linkage^33^.

**Fig. 3:**
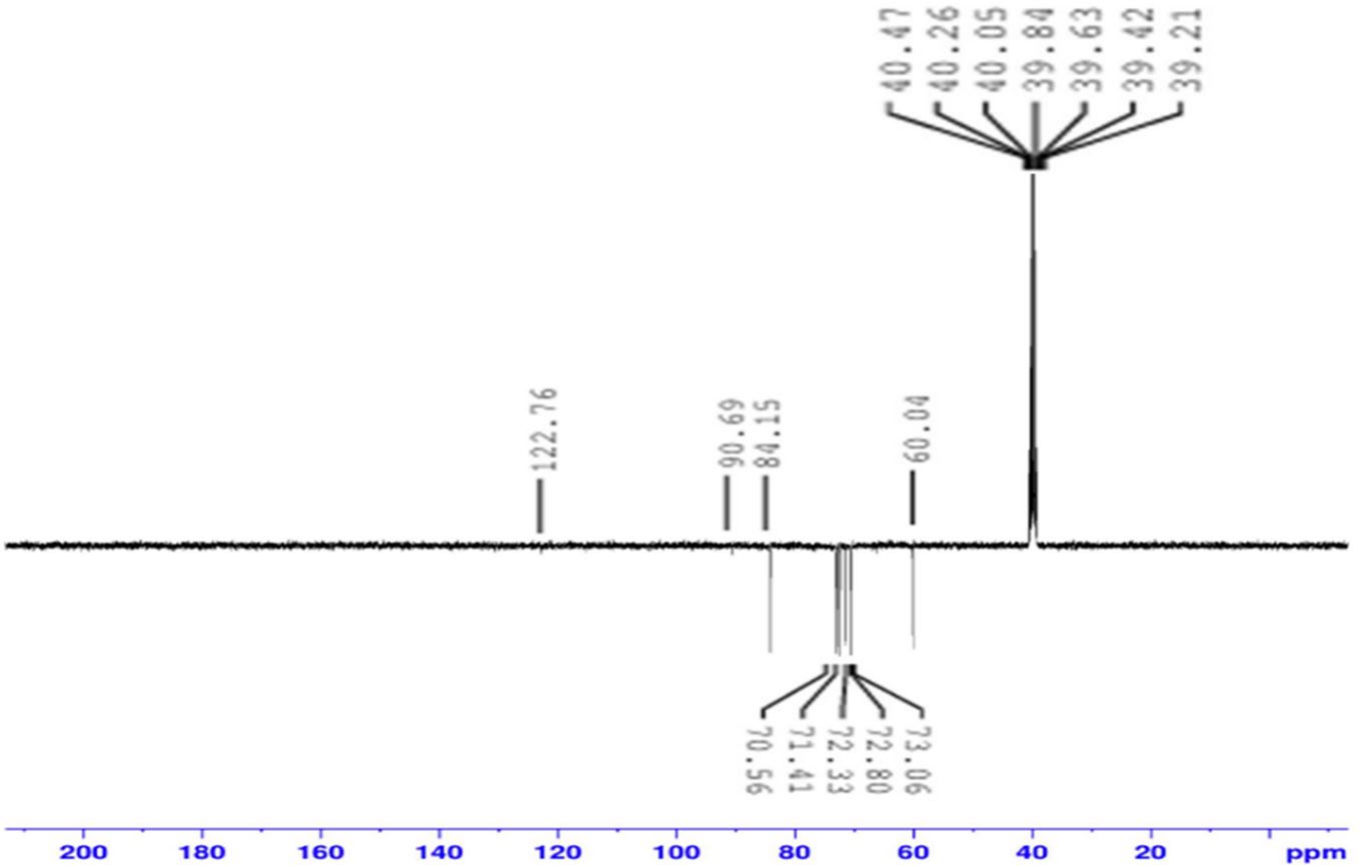
13C NMR spectrum of the synthesized CQDs.

**Fig. 4:**
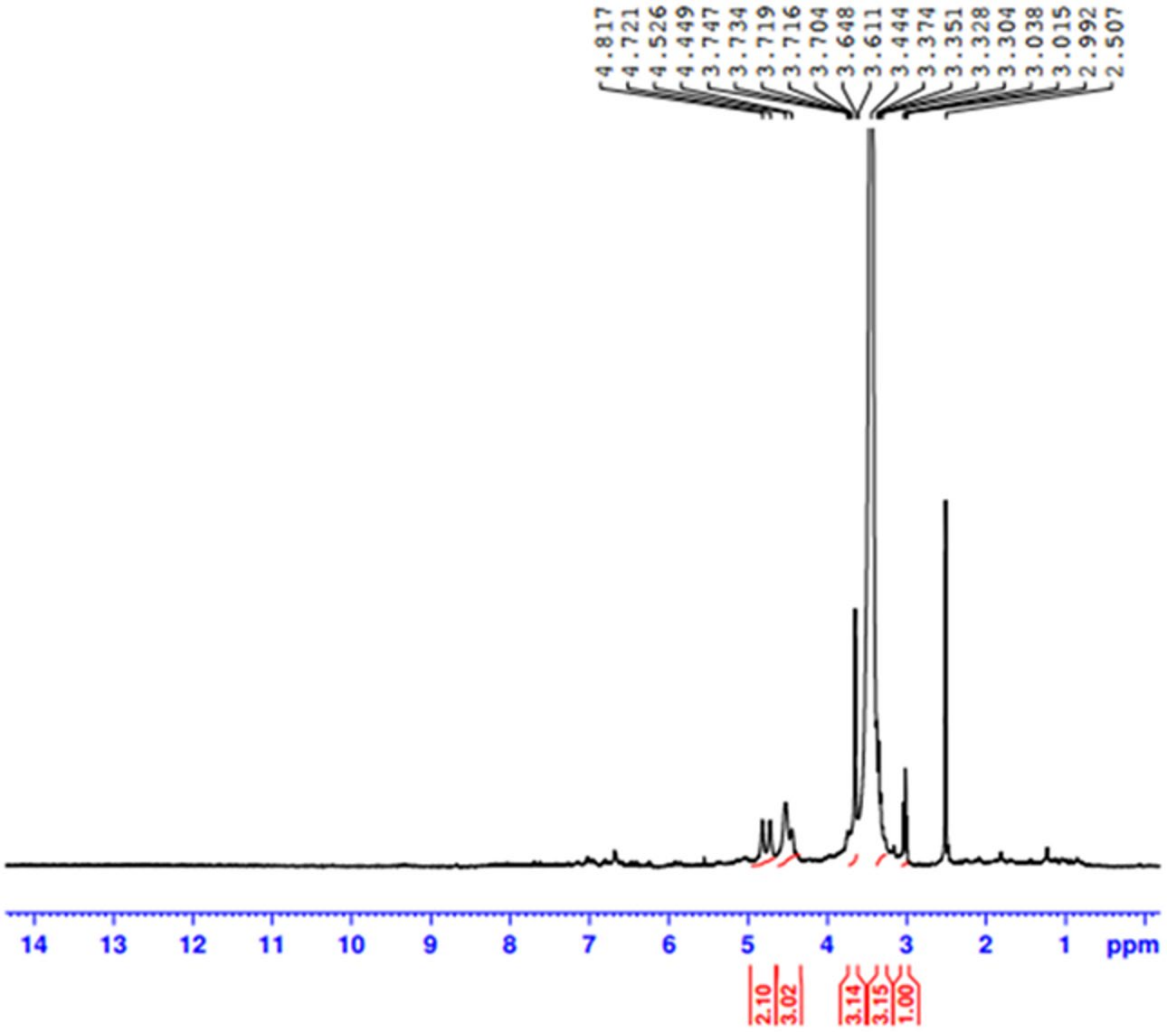
1H NMR spectrum of synthesized CQDs.

**Fig. 5:**
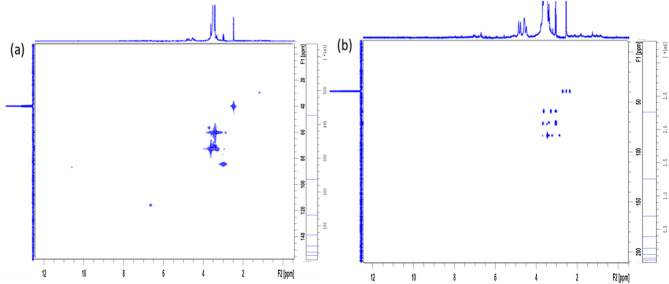
(**a**) HMQC and (**b**) HMBC showing correlation between carbon and proton of CQDs.

**Fig. 6:**
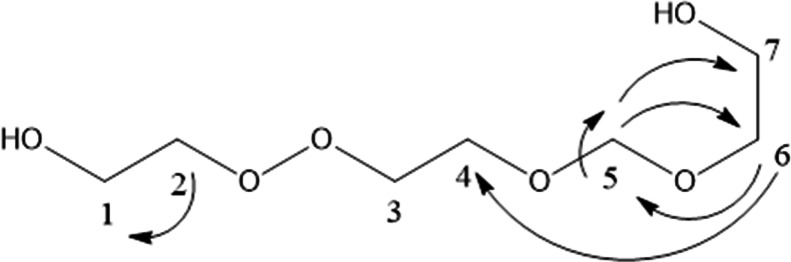
Structure of isolated compound from jojoba leaves after NMR analysis.

ESI–MS (Electrospray Ionization Mass spectroscopy) analysis of the synthesized CQDs in negative mode exhibited molecular ion peaks at m/z = 195 [M–1] and m/z = 239 [M + 2Na–1], corresponding to the molecular formula C_7_H_16_O_6_. In addition, NMR measurements provided detailed structural information. The ^13^C-NMR spectrum (Fig. 3) and ^1^H-NMR spectrum (Fig. 4), including both 1D and 2D experiments (HMQC, and HMBC), played a crucial role in structure elucidation. The ^1^H-NMR spectrum showed overlapping multiple peaks between δ 3.02–3.62, corresponding to the methylene protons (1–7). The ^13^C-NMR spectrum displayed seven negative peaks in the hydroxyl group region, indicating methylene carbons at δ 60.04 (C-1, C-7), δ 70.56 (C-3), δ 71.41 (C-4), δ 72.33 (C-6), δ 72.77 (C-2), and δ 73.06 (C-1), in addition to δ 84.15 (C-5). Some small-intensity peaks appeared in the ^13^C 1D and 2D spectra due to minor impurities. The HMBC and HMQB spectra (Fig. 5a,b) showed cross peaks between (H-5, C-5), (H-6, C-5), (H-7, C-5), (H-5, C-6), (H-4, C-6), and (H-1, C-2). Based on these combined results, the isolated compound of the synthesized CQDs (Fig. 6) was identified as 2-(2-((hydroxyethoxy)methoxy)ethyl)peroxy)ethan-1-ol.

**Fig. 7:**
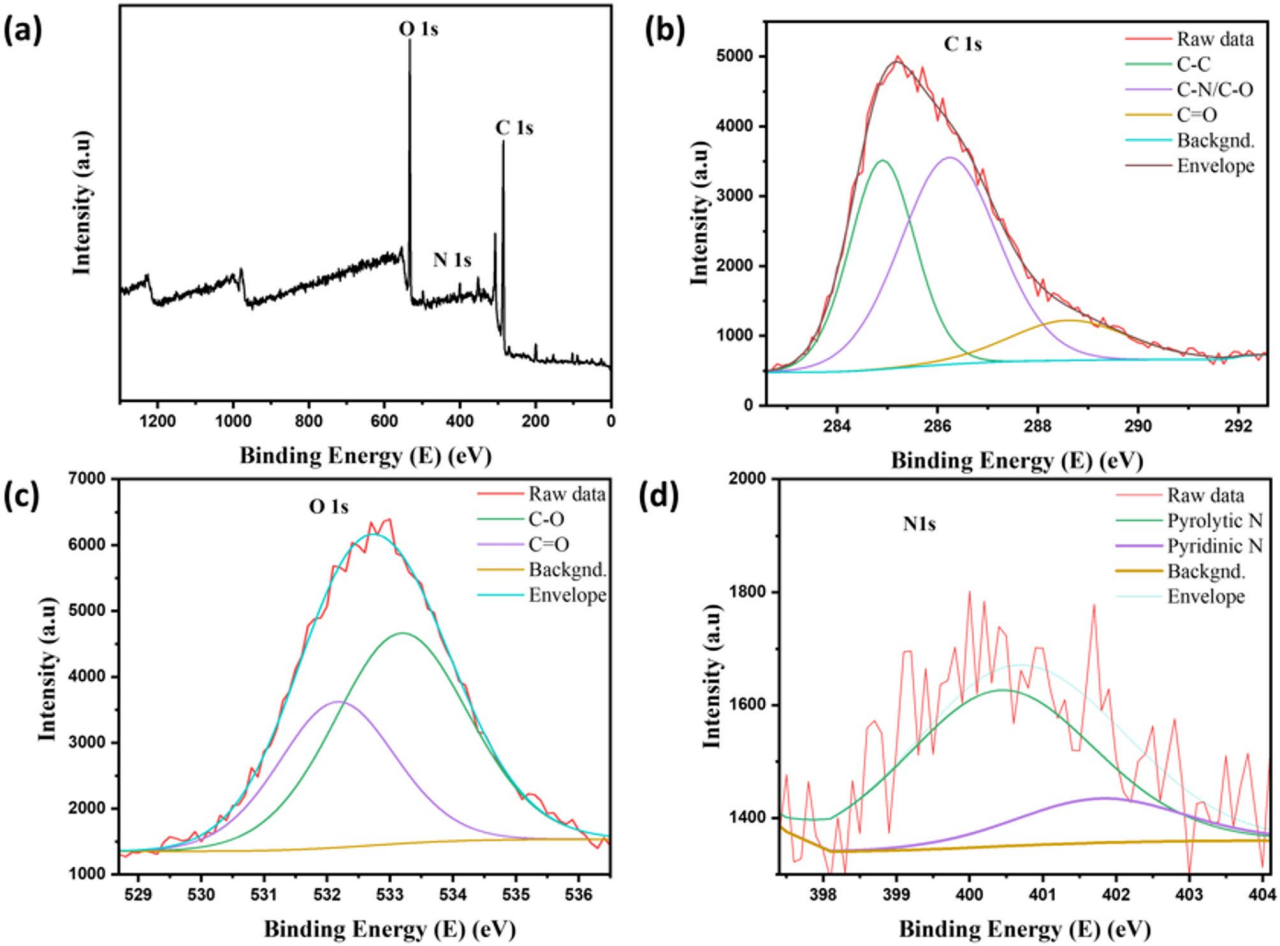
(**a**) XPS survey spectrum of the synthesized CQDs, (**b**, **c**, and **d**), indicates the high-resolution deconvolution of C1s, O1s, and N1s, respectively.

The surface chemical composition and elemental states of the synthesized CQDs were investigated by X-ray photoelectron spectroscopy (XPS), of which multiple distinct peaks spectra were identified at 284.8 eV, 399.8 eV, and 532.9 eV, as shown by survey in Fig. 7a. It is found that the CQDs contain 61.41% of carbon, 4.80% of nitrogen, and 26.72% of oxygen respectively. The respective binding energies for carbon, oxygen, and nitrogen in the survey were deconvoluted as below. A study deconvolution of C1s shown in Fig. 7b indicates three peaks at 284.8 eV, 286.2 eV, and 288.6 eV that are assigned to carbon-to-carbon single bond (C–C), carbon to nitrogen, and carbon to oxygen single bond (C–N/C-O), and carbon to oxygen double bonds (C=O).

Figure 7c shows O1s deconvoluted to two peaks at 532.2 eV and 533.2 eV that can be attributed to C=O and C–O–C functional group, while the high-resolution XPS spectrum of N1s shown in Fig. 7d indicates two peaks at 399.9 eV, 401.8 eV, which correspond to pyrolytic N and pyridinic N that can help to enhance fluorescence quantum yield and can provide localized states within the bandgap which can also help in the facilitation of electron transitions that enhance fluorescence emissions^34^.

Based on these findings, the CQDs might have a nanocrystalline graphitic sp^2^ carbon nucleus functionalized by sp^3^ carbon defects and peripheral carboxyl/carbonyl groups. These studies collectively verify that CQDs have a high concentration of hydrophilic groups on their surface, which contributes to their exceptional water solubility.

### Optical properties of CQDs

**Fig. 8:**
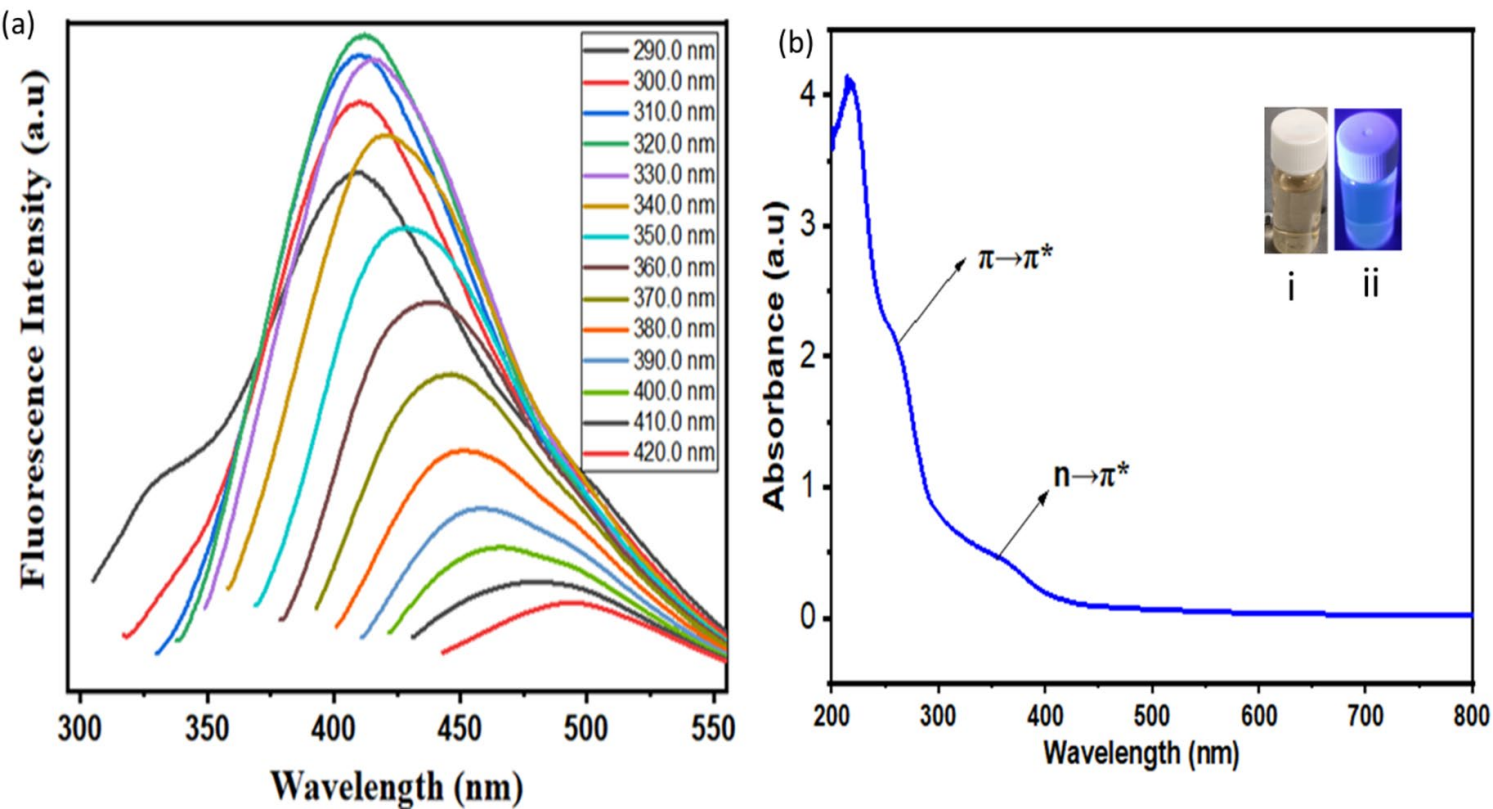
(**a**) The fluorescence spectra of CQDs, (**b**) UV- visible absorption spectrum. The inset in **b** is CQD aqueous solution under (i) normal daylight and (ii) 365 nm UV torch excitation.

The photoluminescence (PL) spectra of the synthesized CQDs were recorded at excitation wavelengths ranging from 290 to 420 nm in 10 nm steps, as shown in Fig. 8a. The emission intensity peaked at 415 nm under 320 nm excitation. A clear excitation-dependent behavior was observed: as the excitation wavelength increased, the emission peak gradually shifted toward longer wavelengths (red shift), while the overall PL intensity decreased at excitations above 330 nm. This behavior is typical of CQDs and is generally linked to the presence of various surface states and size distributions, which create multiple emissive sites. The UV–vis absorption spectrum of the synthesized CQDs was measured across the range of 200–800 nm. Two characteristic peaks appeared at 260 nm and 360 nm, as shown in Fig. 8b. The peak at approximately 260 nm corresponds to the π → π* electronic transition of the conjugated sp^2^ C=C bonds, while the peak at around 360 nm results from the n → π* transitions of C=O groups. The inset images in Fig. 8b(i,ii) display the appearance of the CQDs under daylight and UV illumination at 365 nm, confirming their strong fluorescence capabilities.

**Fig. 9:**
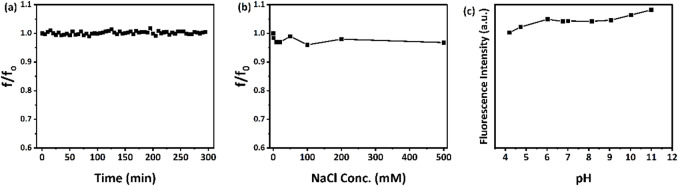
Fluorescence stability against (**a**) time, (**b**), NaCl concentrations, and (**c**) pH environment.

The synthesized CQDs showed excellent fluorescence stability under different environmental conditions. As shown in Fig. 9a, their fluorescence intensity stayed stable over time, which is important for reliable fluorescence-based sensing applications. Additionally, the CQDs remained highly stable in saline environments, as seen in Fig. 9b, where there were negligible changes in fluorescence across NaCl concentrations from 0 to 500 mM. Moreover, the CQDs demonstrated good stability over a wide pH range (4–11), as illustrated in Fig. 9c.

**Fig. 10:**
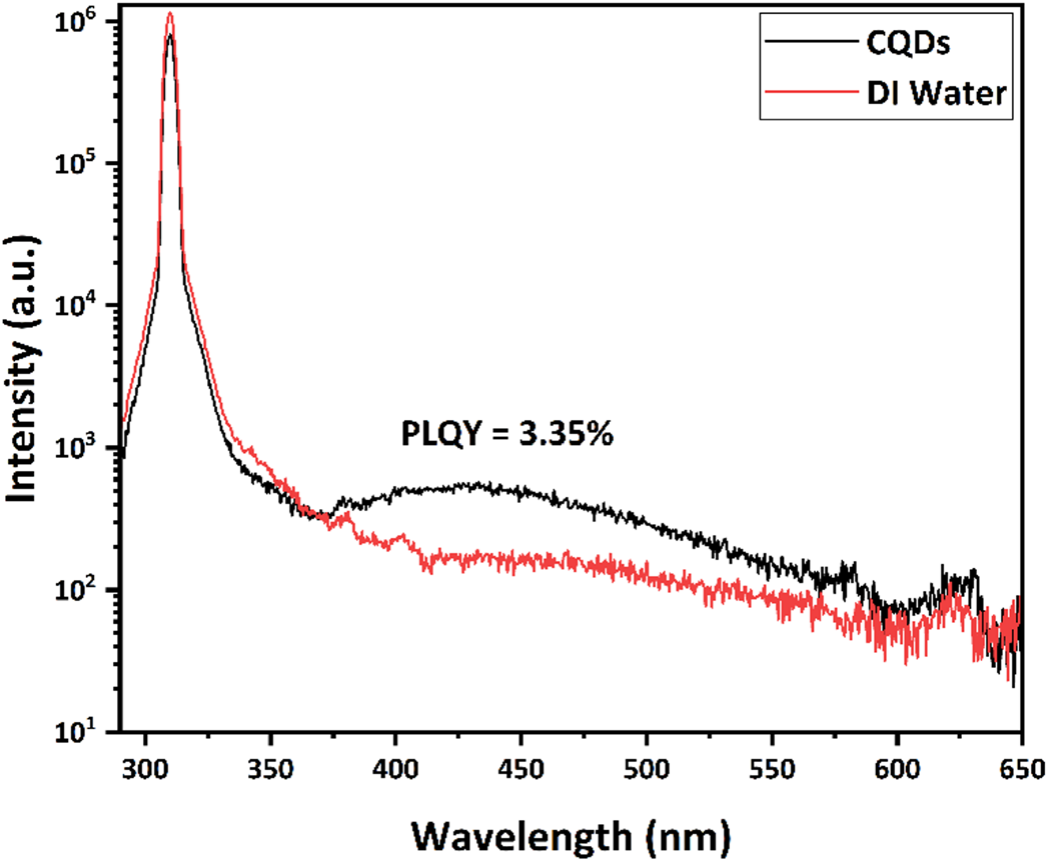
Fluorescence spectra for CQDs and its solvent.

The QY of the synthesized CQDs was determined using an absolute quantum yield measurement system (Fig. 10), yielding a value of 3.35%. Although relatively modest compared to chemically engineered CQDs, this value is consistent with many plant-derived CQDs and highlights the effectiveness of jojoba leaves as a sustainable precursor.

**Fig. 11:**
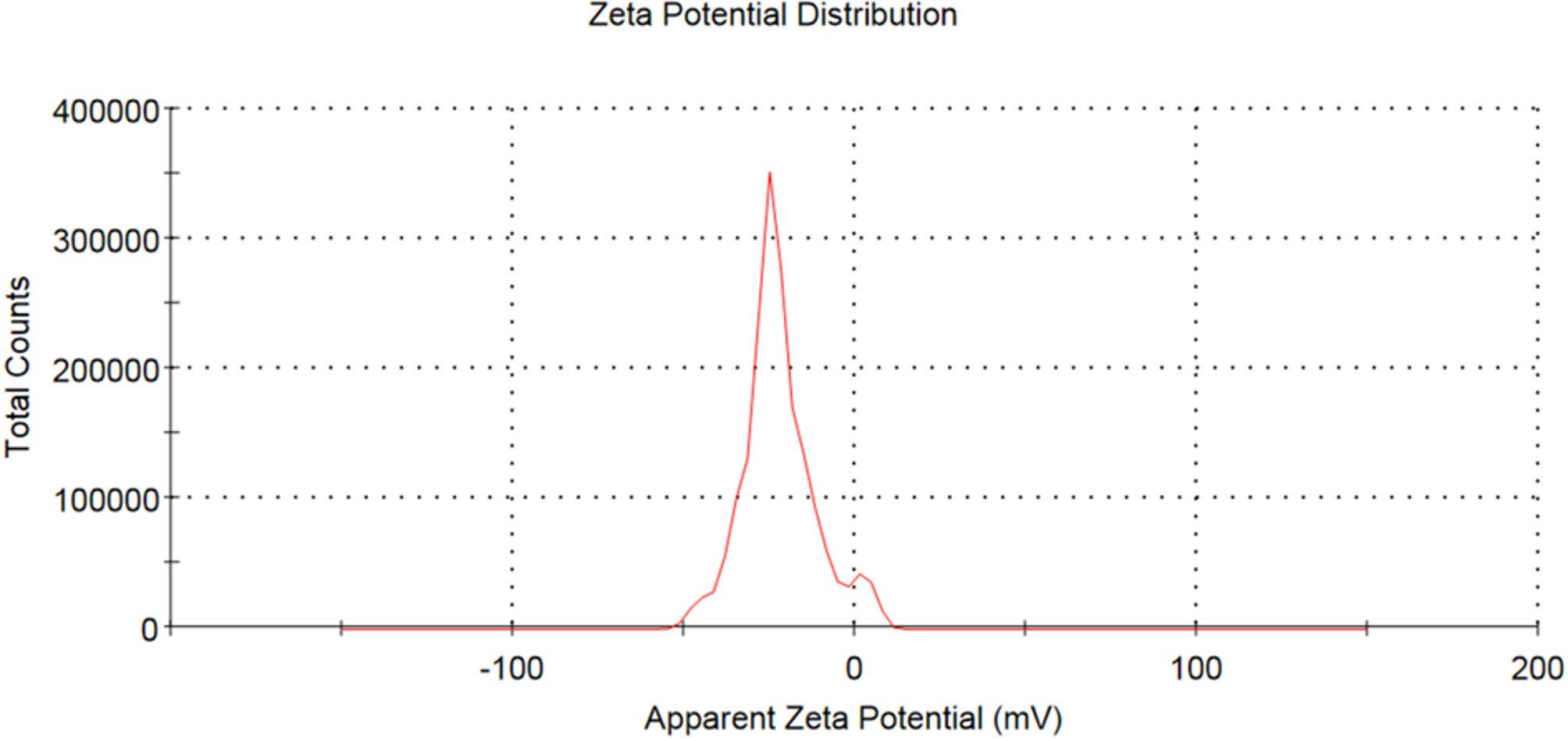
Zeta potential spectrum of CQDs.

Figure 11 shows the Zeta potential spectrum for the synthesized CQDs with a potential value of − 22.0 mV, indicating moderate electrostatic repulsion between particles. This value demonstrates that CQDs remain stable enough for sensing applications within the timescales tested in this study^35^.

### Optical properties of CQDs

**Fig. 12:**
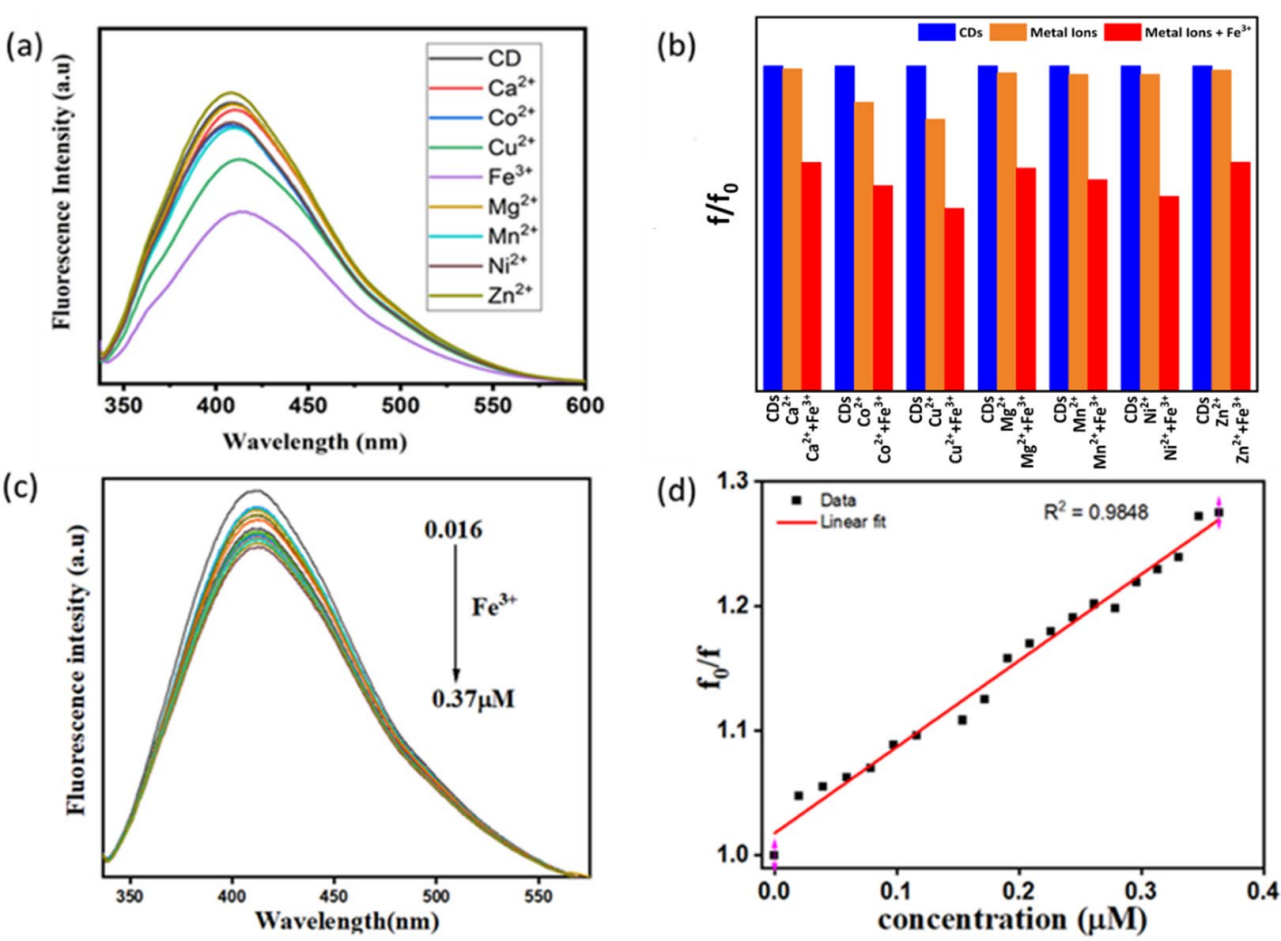
(**a**) selectivity fluorescence intensity of CQDs in the absence and presence of different metal ions, (**b**) Fluorescence intensity of F_0_/F against mixed metal ions in CQDs, (**c**) the fluorescence spectra of the sensor experiment with different concentration.

The fluorescence selectivity of the synthesized CQDs toward various metal ions (Ca^2+^, Co^2+^, Cu^2+^, Mg^2+^, Mn^2+^, Fe^3+^, Ni^2+^, and Zn^2+^) was evaluated by monitoring their effect on the fluorescence intensity. For each test, 50 µL of a 4 µM metal ion solution was added to 2 mL of CQDs. The initial fluorescence intensity of the CQDs without metal ions was denoted as ${F}_{0}$, while f represents the intensity after ion addition, with excitation at 320 nm. Among the tested ions, Fe^3+^ caused a pronounced quenching of the CQD fluorescence, whereas the other ions produced negligible effects, as illustrated in Fig. 12a. Moreover, a quantitative selectivity test was performed to further evaluate the preferential interaction of CQDs with Fe^3+^ ions in the presence of competing metal ions. As shown in Fig. 12b, the fluorescence intensity of the CQDs in mixed-ion solutions remained strongly quenched only in the presence of Fe^3+^, while the influence of other metal ions was negligible. This result confirms that the CQDs exhibit a strong affinity and high selectivity toward Fe^3+^ even under competitive conditions.

To investigate the sensitivity and linearity range of our CQDs towards the analyte, we prepared different concentrations of Fe^3+^ ion using the same concentration (4 µM), and a similar technique by adding 5 µL to 2 mL of CQDs, and each time, fluorescence intensity is checked. It’s noticed that the fluorescence intensity of the CQDs decreases as the concentrations of Fe^3+^ ions increase in the sample, as shown in Fig. 12c, following a linear relationship expressed by $y=3.07x+0.99$. Then, this relationship between the Fe^3+^ ion concentrations and the fluorescence intensity ratio ${F}_{0}/F$ at the range of 0.016–0.37 µM with R^2^ = 0.985 was found, as shown in Fig. 12d, and according to the Stern–Volmer equation ${F}_{0}/F$= $1+{K}_{sv}[Q]$, where $F$ and ${F}_{0}$ are the fluorescence intensities of the CQDs with and without Fe^3+^, respectively, ${K}_{sv}$ is the quenching constant, while Q is the Fe^3+^ concentration.

The limit of detection (LOD) = 3σ/s, where s is the linear fit slope and σ is the standard deviation, was found to be 0.018 µM. This value is the smallest obtained so far for Fe^3+^ ion detection using biomass-derived nanomaterials and demonstrates that our CQDs represent one of the most sensitive fluorescence-based detection methods for Fe^3+^ compared to other reports in the literature (see Table 1). It is worth noting, however, that biomass-derived CQDs in general also demonstrate relatively high limits of detection in some cases, highlighting the significance of achieving such an ultra-low value in this work.

**Table 1:** Comparison of CQDs-based sensor for Fe^3+^ detection.

Sensor	Synthesis methods	Linear range (µM)	LOD (µM)	References
Catharanthus roseus leaves	Hydrothermal/carbonization	1–6	0.8	^1^
Black plum	Hydrothermal	0–80	0.13	^2^
CQDs from Tagetes patula flower	Hydrothermal	0–4	0.32	^3^
N-CQDs from watermelon juice	Hydrothermal/carbonization	0–300	0.16	^4^
Ophiopogon japonicus leaves	Hydrothermal	10–60	1.15	^5^
Carbon dots from honey	Hydrothermal	0.005–100	0.0017	^6^
GQDs from biomass waste	Pyrolysis/ Microwave	0–50	2.5	^36^
N-CQDs from aminobenzoic acid	Hydrothermal	0–1.6	0.05	^37^
CQDs from orange peels	Chemical treatment	0–400	0.2	^38^
CQDs from jojoba leaves	Hydrothermal	0.016–0.37	0.018	Current work

### Real sample analysis

To assess practical applicability and the feasibility of the synthesized CQD-based sensor as a probe for Fe^3+^ detection, a sample of tap water, collected from the Energy Materials Lab, Egypt-Japan University of Science and Technology (E-JUST), Egypt. The water sample underwent filtration through a 0.22 mm membrane to eliminate any potential interfering substances that may interact with the CQDs. Following the same procedure by adding (20, 40, 60, and 80) µL of prepared concentration (4 µM) of Fe^3+^ ion into 2 mL of CQDs and 1 mL of tap water, the added concentrations of Fe^3+^ were calculated using the dilution equation and their corresponding found concentrations from the calibration curve, (see Table 2). The recovery rates are within the range of (80.1–114.0) %, and the relative standard deviation (RSD) for the triplicate measurements falls below 5%. These findings suggest that this sensor method is effective for the detection of Fe^3+^ in tap water.

**Table 2:** Detection of Fe^3+^ ion in tap water and RSD (N = 3).

Sample	Fe^3+^ added (μM)	Fe^3+^ found (μM)	Recovery (%)	RSD (%)
Tap water	0.0265	0.0302	114.0	0.22
	0.0526	0.0421	80.5	0.17
	0.0784	0.0841	107.5	0.23
	0.1039	0.1038	99.9	0.33

### Mechanism of CQDs fluorescence quenching by Fe^3+^

**Fig. 13:**
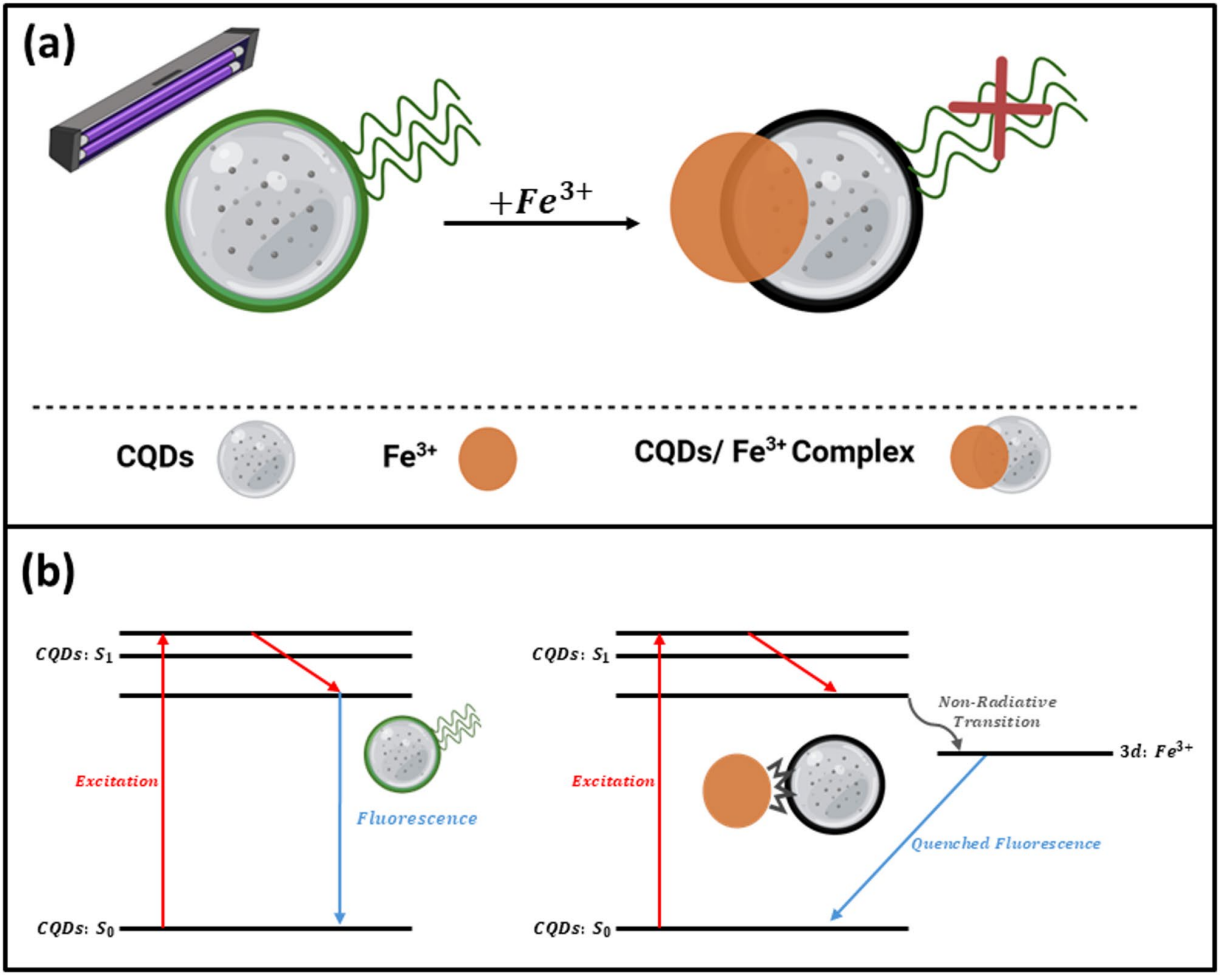
Schematic diagram for the interaction of CQDs with Fe^3+^.

Fluorescence quenching of CQDs by Fe^3+^ primarily arises from strong interactions between Fe^3+^ ions and oxygen- or nitrogen-containing surface groups on the CQDs, such as carboxyl, hydroxyl, and particularly phenolic –OH functionalities. These groups can chelate Fe^3+^ through inner-sphere coordination, providing multiple oxygen donor sites and a polar environment that favor complexation. The predominance of oxygen donor sites on the CQD surface accounts for the high selectivity toward Fe^3+^ (Fig. 12b). As a result, stable non-emissive CQD–Fe^3+^ complexes are formed, leading to fluorescence quenching via a static mechanism. In this case, the population of emissive species decreases while the excited-state lifetime of the remaining fluorophores remains unchanged, as illustrated in Fig. 13a.

One the other hand, dynamical quenching which involve the interaction between the excited state CQDs and the ferric ions resulting in fluorescence quenching. Figure 13b shows that Fe^3+^ has significant potential to quench the FL by energy or electron transfer from an excited state to its 3d orbital owing to its paramagnetic behavior^39,40^. In particular, the oxygenated groups on the CQDs can donate lone pairs to the 3d orbitals of Fe^3^^+^, facilitating complexation and electron transfer^37^. Upon excitation of the CQDs, electron–hole (e^−^/h^+^) pairs are generated. The excited electrons can then transfer to the Fe^3+^ 3d orbitals, suppressing radiative recombination and thereby quenching fluorescence, as shown in Fig. 13b.

**Fig. 14:**
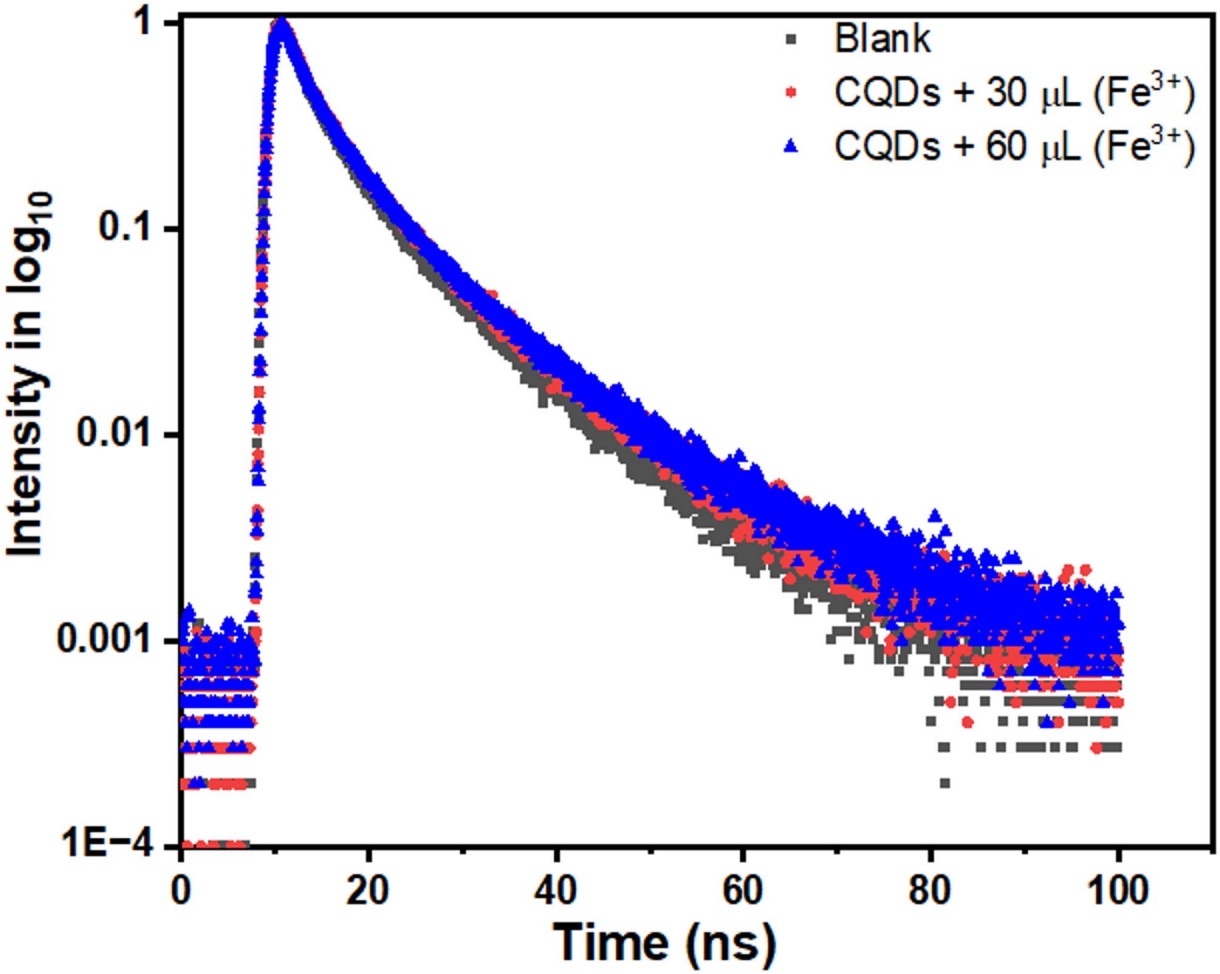
Fluorescence lifetime decay curves of CQDs in the absence of Fe^3+^, as well as in the presence of different volumes of Fe^3+^ ions.

To probe this quenching mechanism in greater detail, time-resolved photoluminescence (PL) measurements were carried out in the absence and presence of Fe^3+^ ions. Samples have excited using 340 nm picosecond pulsed LED, and the fluorescence decay profiles have been fitted according to Eq. (1)

1 $$\begin{array}{*{20}c} {Y = A + B_{1} e^{{ - \frac{t}{{\tau_{1} }}}} + B_{2} e^{{ - \frac{t}{{\tau_{2} }}}} + B_{3} e^{{ - \frac{t}{{\tau_{3} }}}} } \\ \end{array}$$

where τ_1_, τ_2_, and τ_3_ represent distinct decay components; A is the baseline value; and B_1_, B_2_, and B_3_ are the corresponding amplitudes. The PL decay curves are presented on Fig. 14.

The calculated lifetimes, along with the average lifetime $\left( {\tau_{avg} } \right)$, are summarized in Table 3 for different Fe^3+^ concentrations (30 and 60 µL). The average lifetime was calculated using Eq. (2)^41^

2 $$\begin{array}{*{20}c} {\tau_{avg} = \frac{{B_{1} \tau_{1}^{2} + B_{2} \tau_{2}^{2} + B_{3} \tau_{3}^{2} }}{{B_{1} \tau_{1} + B_{2} \tau_{2} + B_{3} \tau_{3} }}} \\ \end{array}$$

**Table 3:** CQDs Fluorescence lifetimes decay analysis.

Synthesis CQDs	Different lifetimes (ns)	Average lifetimes (ns)
CQDS	${\tau }_{1}=$ 1.988 (${\text{B}}_{1}=0.0308)$	$7.9 \pm 0.2$
	${\tau }_{2}=$ 6.327 (${\text{B}}_{2}=0165)$	
	${\tau }_{3}=$ 17.75 (${\text{B}}_{3}=0.0030)$	
CQDs + 30 µL Fe^3+^	${\uptau }_{1}=1.982 ({\text{B}}_{1}=0.0303)$	$8.1\pm 0.2$
	$${\tau }_{2}=6.353 \left({\text{B}}_{2 }= 0.0169\right)$$	
	$${\tau }_{3}=17.72 ({\text{B}}_{3}=0.0033)$$	
CQDs + 60 µL Fe^3+^	${\uptau }_{1}=2.038 ({\text{B}}_{1}= 0.0306)$	$8.3\pm 0.2$
	$${\uptau }_{2}= 6.652 \left({\text{B}}_{2}=0.0167\right)$$	
	$${\tau }_{3}= 18.47 ({\text{B}}_{3}=0.0031)$$	

The results show that ${\tau }_{avg}$ remains almost unchanged with increasing Fe^3+^ concentration, confirming that the interaction follows a static quenching mechanism^42^. In this case, Fe^3+^ ions form stable ground-state complexes with CQD surface groups, particularly through direct interactions with phenolic hydroxyl groups on the CQD surface, rather than quenching fluorescence through collisional encounters. This strong binding underlies both the high selectivity and the ultrasensitive detection achieved, since competing ions are less likely to form such stable complexes^38^. However, static quenching may limit sensor reusability if the CQD–Fe^3+^ complexes are too stable. In contrast, dynamic quenching caused by collisional interactions typically shortens the excited-state lifetime, is reversible, but often less selective and highly dependent on environmental conditions such as temperature and solvent viscosity. Thus, identifying the quenching pathway provides valuable insights for designing and optimizing CQD-based sensors^42,43^.

## Conclusion

We have successfully demonstrated a facile and environmentally friendly one-pot hydrothermal synthesis of CQDs from abundant jojoba leaves, underscoring both the sustainability of this approach and its potential scalability for cost-effective production. The resulting CQDs, with an average diameter of 3.5 nm, exhibited blue fluorescence with a quantum yield of 3.35%. These CQDs proved effective for Fe^3+^ ion detection, showing a significant fluorescence quenching response with an ultra-low detection limit of 0.018 µM. The quenching mechanism was confirmed to be static, as evidenced by the unchanged fluorescence lifetime decay of the quenched emission, highlighting their potential as ultrasensitive optical probes for Fe^3+^. This green synthesis strategy also opens avenues for exploring other plant-derived precursors in the development of CQDs, broadening their applicability in areas such as biomedical imaging and industrial monitoring.

## Data Availability

All data generated or analyzed during this study are included in this published article.
